# Modeling the consumer’s perception of experiential marketing in the Romanian private ophthalmologic services

**DOI:** 10.22336/rjo.2017.40

**Published:** 2017

**Authors:** Consuela-Mădălina Gheorghe, Iuliana-Raluca Gheorghe, Victor Lorin Purcărea

**Affiliations:** * Department of Marketing and Medical Technology, “Carol Davila” University of Medicine and Pharmacy, Bucharest, Romania

**Keywords:** consumer perception, experiential marketing, private ophthalmologic services, word-of-mouth communication

## Abstract

**Introduction.** The importance of experience in marketing grew, as the concept itself is very personal and difficult to measure. Experience turns out to be complicated but once placed in a context it gets significant features. As the health care competitive environment increases, marketers are looking for new and effective methods of engaging consumers by using experiential marketing strategies. Moreover, little is known about the consumers’ perceptions related to ophthalmologic services.

**Aim.** The objective of this paper was to measure the consumer’s perception of experiential marketing in the Romanian private ophthalmologic services by using structural equation modeling.

**Materials and Methods.** The Experiential Marketing model consisted of the following components: Sense Experience, Feel Experience, Think Experience, Act Experience and Relate Experience as well as the consequences of applying Experiential Marketing in the form of willingness to purchase a service, generating word-of-mouth communication and building consumer loyalty.

The sampling method was non-probabilistic, using the snowball technique and the sample was made up of 190 people who wore eyeglasses for more than 3 years.

The instrument for data collection was a self-administered questionnaire, which consisted of 2 parts: the first section contained several demographic questions and the second section encompassed closed end questions related to the perception of private ophthalmologic services from an experiential marketing perspective. All the second section questions were measured on a 5-point Likert scale ranging from 1 with Strongly Disagree to 5 to Strongly Agree. The data analysis was conducted in SPSS and the structural equation modeling was performed in WarpPLS version 6.0.

**Findings.** There were 71.05% respondents, who appreciated the application of experiential marketing in private ophthalmologic services, followed by 18.95%, who were confused. The demographic profile of respondents encompassed the following features: females with the ages between 36 and 45, from the rural area and with a middle level of education, their private ophthalmologic consultation frequency was at every 3 months and they also declared having a stable physician.

Going further with the analysis, 89.63% of the respondents admitted they were willing to buy a private ophthalmologic service based on the experiential marketing application strategies.

The design of a model containing both the constituent elements of the experimental marketing and its consequences in ophthalmologic services was conducted by modeling with structural equations in WarpPLS version 6.0 software. Thus, the validity of the model was assessed with the Cronbach’s alpha coefficients, Composite reliability values, as well as with the Average Variance Extracted coefficients, and the fitness of the model was determined by using the ARS, APC, and AVIF values, respectively.

According to the beta coefficients and levels of statistical significance (p<0.05), some hypotheses have been rejected or negative relationships have been established between dependent and independent variables.

**Conclusions.** Sense experience had a negative impact on WOM and consumer loyalty, Think Experience had a negative influence on the WOM, Relate Experience had a negative impact on consumer Loyalty, as well as Relate Experience had a negative impact on willingness to purchase an ophthalmologic service. In contrast, the following positive relationships were established: Feel Experience established a positive relationship with WOM and consumer loyalty, Think Experience presented a positive impact both on consumer loyalty and on willingness to purchase an ophthalmologic service, Act Experience presented a positive impact on WOM and willingness to purchase and last, willingness to purchase an ophthalmologic service presented a positive influence on consumer loyalty and WOM. However, managers in Ophthalmology can successfully use experimental marketing strategies if they promote a story, meaning a life experience.

## Introduction

Experience has played a core role in health care services, as today most global economies are related to the consumption of experiences. Moreover, the importance of experience in marketing grew, as the concept itself is very personal and difficult to measure. Clearly, experience turns out to be complicated but placed in a context it gets a significant feature as for example an interaction between a physician and a patient. Many specialists consider experience to be subjectively assessed as it involves a wide range of feelings and sensorial stimuli and it implies involvement at different levels [**1**].

As the health care competitive environment increases, marketers are looking and trying to find new and effective methods of engaging consumers. 

The concept of service itself has also evolved from being considered commercial to being something that defines a lifestyle [**2**]. The process of purchasing a service has also led to changes in consumer’s behavior, namely, consumers are becoming more educated and demanding. In the same vein, Lindstrom stated that most people would always want to experience touch, smell, sound, and taste, as well as visual appeal, before they buy a service, as it helps them tangibilize it [**3**]. At the same time, consumers have begun to frequently establish relationships between themselves and the service they desire [**4**]. Health care services are no exception from these features. Further, this is where, more and more often, the interest of marketing specialists for experiential marketing grows, in order to provide the service experiences that awaken all their senses and offer them a unique sensation and will remain engrained in their minds for a long period of time [**5**].

According to Kotler [**6**], there are two types of marketing: traditional marketing and modern marketing. As far as modern marketing is concerned, it overtook the traditional approach, as it focuses on both customer experience and experiential marketing. Moreover, Holbrook [**7**] believed that when organizations start implementing experiential marketing principles, the major focus would turn from service performance to experiences, as illustrated in **Table 1**. 

**Table 1:** Traditional marketing versus modern marketing [**8**]

	Traditional Marketing	Modern Marketing (Experiential Marketing)
Focus	Service features and benefits	Consumer experience
Scope	Narrow definition of service categories and consumption	Broader consumption situation and socio-cultural context
The consumer model is based on	Rational decisions	Rational and emotional decisions
Marketer’s Approach	Analytical, verbal and quantitative	Eclectic, verbal, visual and intuitive

The main objective of this paper was to measure the consumer’s perception of experiential marketing in the Romanian private ophthalmologic services by using structural equation modeling. As such, the following objectives have been settled:

- Identifying the demographic profile of the respondents who appreciate the application of experiential marketing in private ophthalmologic services. 

- Determining the proportion of consumers who have the willingness to buy a private ophthalmologic service after they appreciate the application of experiential marketing principles. 

- Elaborating a model that will encompass both the elements of experiential marketing as well as the consequences of applying its principles in private ophthalmologic services. 

**Conceptual framework**

a. Experiential marketing 

The idea of experiential marketing relies on the assumption that the main strategic policies and marketing decisions concentrate not only on supply, but also on the consumption and the experience that can be shaped based on designing an entire physical environment and an operational process for its consumers to experience [**9**].

The vast majority of scientific literature on experiential marketing focuses on the following dimensions, which are known as strategic experiential modules (SEMs) [**10**]:

- Sense is the dimension which refers to the experience as perceived through the five human senses;

- Feel dimension is concerned with the consumer’s emotional engagement but how to trigger certain positive emotions in consumers may be difficult as there are differences between consumption cultures;

- Think dimension is linked to the cognitive elements of the service’s features and attributes. It relates to stimulating the service consumption by using technology and innovativeness and make consumers associate it with something different;

- Act dimension refers to consumer’s actions and their physical engagement;

- Relate dimension concentrates on the social dimension of the experience, meaning that the experience allows the consumer, through the process of consumption, to establish ties with various peers and communities. 

However, most Schmitt’s work (1999) focused on the following key features of experiential marketing:

- Consumer experience should replace the functional values of services with sensory, emotional, cognitive, behavioral and relational values;

- The consumption experience should be adjusted to the socio-cultural context;

- Consumers should be considered both as rational and emotional beings and so, the consumption experience is often driven by emotions, fantasies and creativity;

- Methods and tools are eclectic, meaning that experiential marketing offers specialists the opportunity to address the consumption experience from different perspectives by using both analytical and intuitive methods. 

b. Experiential marketing and consumer loyalty

 Bowen and Chen (2011) [**11**] define consumer loyalty as the degree to which a consumer shows repeated purchasing behavior of a service as well as has a positive attitudinal behavior towards the provider. Moreover, Oliver (1999) [**12**] defines loyalty as a deeply held commitment to rebuy a preferred service in the future, despite being influenced by situational factors. In addition, experiential marketing seeks to identify behaviors and attitudes in order to add value to the interaction between the provider and the consumer, by delivering a certain value. The idea that stands behind is that once the consumer follows and appreciates that set of values, he/ she is more likely to become a loyal consumer of that service. Similarly, Schmitt (1999) mentioned that creating a pleasant experience for consumers is the key to consumer loyalty and Chen and Lee showed that increased efforts in sense, feel and think components of experiential marketing can lead to higher levels of consumer loyalty [**13**]. 

c. Experiential marketing and word-of-mouth communication (WOM)

Satisfaction is the degree to which consumer expectations related to the consumption of a service are fulfilled and is the interaction between expectations and performances (Oliver, 1999). Moreover, satisfaction may be defined as being an experience-based attitude, meaning that the consumer’s evaluation depends on the individual experience with the service [**14**]. Kim et al. reached the conclusion that satisfied consumers provide positive recommendations to peers and their family members in the shape of word-of-mouth communication [**15**].

d. Experiential marketing and willingness to purchase 

It is acknowledged that experiential marketing is the exogenous key construct materialized in willingness to purchase as well as purchasing behavior. Moreover, consumers produce familiarity, relationship, and closeness after experiencing the consumption and which result in an increased consumer intention to purchase a service [**16**].

**Proposed Conceptual Framework**

**Fig. 1:**
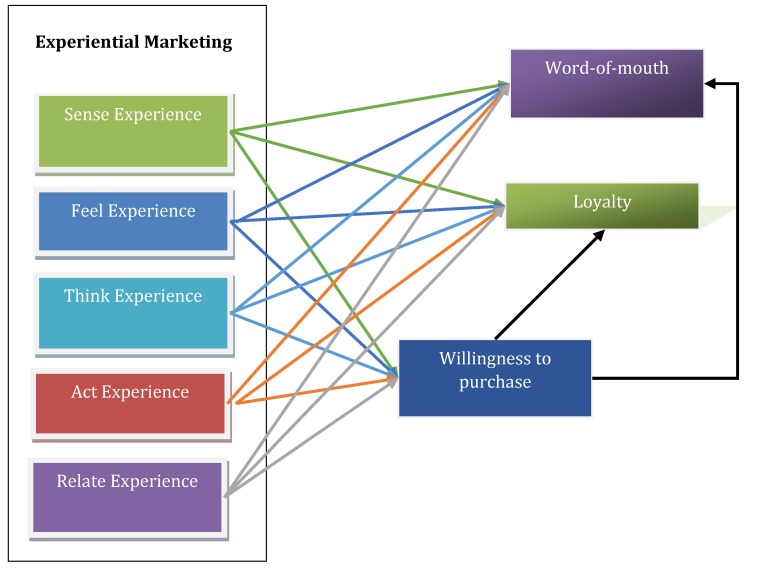
The proposed conceptual framework

The proposed conceptual framework for the model is illustrated in **Fig. 1** and the hypotheses of the model in **Table 2**. 

**Table 2:** The hypotheses of the model

No.	Hypotheses
H1	Sense Experience is positively related to the WOM communication in private ophthalmologic services.
H2	Sense Experience is positively related to consumer loyalty in private ophthalmologic services.
H3	Sense Experience is positively related to the willingness to purchase a private ophthalmologic service.
H4	Feel Experience is positively related to the WOM communication in private ophthalmologic services.
H5	Feel Experience is positively related to consumer loyalty in private ophthalmologic services.
H6	Feel Experience is positively related to the willingness to purchase a private ophthalmologic service.
H7	Think Experience is positively related to the WOM communication in private ophthalmologic services.
H8	Think Experience is positively related to consumer loyalty in private ophthalmologic services.
H9	Think Experience is positively related to the willingness to purchase a private ophthalmologic service.
H10	Act Experience is positively related to the WOM communication in private ophthalmologic services.
H11	Act Experience is positively related to consumer loyalty in private ophthalmologic services.
H12	Act Experience is positively related to the willingness to purchase a private ophthalmologic service.
H13	Relate Experience is positively related to the WOM communication in private ophthalmologic services.
H14	Relate Experience is positively related to consumer loyalty in private ophthalmologic services.
H15	Relate Experience is positively related to the willingness to purchase a private ophthalmologic service.
H16	The willingness to purchase a private ophthalmologic service is positively related to WOM communication.
H17	The willingness to purchase a private ophthalmologic service is positively related to consumer loyalty.

## Material and methods 

The sampling method was non-probabilistic, using the snowball technique and the sample was made up of 190 people who wore eyeglasses for more than 3 years.

The instrument for data collection was a self-administered questionnaire which consisted of 2 parts: the first section contained several demographic questions and the second section encompassed closed end questions related to the perception of private ophthalmologic services from an experiential marketing perspective through its components. All the second section questions were measured on a 5-point Likert scale ranging from 1 with Strongly Disagree to 5 to Strongly Agree, as shown in **Table 3** but modified for measuring a health care service. 

**Table 3:** The questionnaire design

Construct	Measurement	No. of items	Source
Experiential Marketing	Sense Experience	7	Schmitt (1999)
	Feel Experience	7	Schmitt (1999)
	Think Experience	4	Gentile et al. (2007) [**17**]
	Act Experience	5	Maghnati et al. (2012) [**18**]
	Relate Experience	3	Schmitt (1999)
Consumer loyalty		4	Chen et al. (2009) [**19**]
			Chou et al. (2010) [**20**]
WOM		2	-
Willingness to purchase		3	Soderlund and Ohman (2005) [**21**]

The data analysis was conducted in SPSS and the modeling was performed in WarpPLS version 6.0. More exactly, the Structural Equation Modeling was used to test the measurement and the structural design of the model as well as the research hypotheses presented in **Table 2**. 

**Findings**

There were 71.05% respondents, who appreciated the application of experiential marketing in private ophthalmologic services, followed by 18.95%, who were confused. The demographic profile was that the respondents were females with the ages between 36 and 45, from the rural area and with a middle level of education. Moreover, their private ophthalmologic consultation frequency was at every 3 months and they also declared having a stable physician. 

Going further with the analysis, 89.63% of the respondents admitted they were willing to buy a private ophthalmologic service based on the experiential marketing application. 

- The structural equation model 

a. Reliability measurement

A review of **Table 4** and **Table 5** shows several results concerning the reliability in a PLS measurement model. As such, **Table 4** illustrates the Cronbach’s alpha coefficients and the composite reliability coefficients. The cutoff point for these 2 types of coefficients is recommended to be more than 0.7, however, values of 0.6 were also admitted. The average variance extracted should exceed the suggested value of 0.5 for all measures [**22**]. Taking into consideration the results in **Table 4**, it was concluded that the internal consistency of the model was assessed.

**Table 4:** The internal reliability of the model

Construct	Cronbach’s Alpha Coefficient Values	Composite Reliability (CR)	Average Variance Extracted (AVE)
Sense Experience	0.86	0.91	0.72
Feel Experience	0.81	0.89	0.73
Think Experience	0.92	0.96	0.92
Act Experience	0.87	0.91	0.73
Relate Experience	0.83	0.92	0.86
Willingness to purchase	0.72	0.84	0.74
Word-of-Mouth Communication	0.72	0.88	0.78
Consumer Loyalty	0.75	0.86	0.77

In **Table 5**, the Pattern Matrix confirms the existence of 8 dictinct factors that are the equivalents of the 8 latent constructs of experiential marketing. In addition, the resulted matrix also confirmed that not all the items were still included in the model. However, the resulted Pattern Matrix from the WrapPLS analysis supports the fact that the selection of variables was optimal.

**Table 5:** The Pattern Matrix of the Model

	sense	affect	think	act	rel	int	wom	loi
es_it1	0.781	-0.050	-0.154	0.083	0.020	0.131	-0.152	0.086
es_it3	0.849	-0.003	0.001	-0.018	-0.061	-0.014	0.042	-0.109
es_it4	0.890	0.031	0.047	-0.018	0.013	-0.042	0.040	0.060
es_it5	0.870	0.017	0.091	-0.040	0.027	-0.063	0.057	-0.034
ea_it1	-0.058	0.809	-0.050	0.048	-0.112	0.035	0.041	0.040
ea_it3	0.098	0.869	-0.042	-0.069	0.022	-0.060	-0.019	0.019
ea_it4	-0.041	0.893	0.087	0.022	0.082	0.025	-0.020	-0.055
eg_it3	0.032	-0.023	0.963	0.014	-0.018	0.018	0.025	0.007
eg_it4	-0.032	0.023	0.965	-0.014	0.018	-0.018	-0.025	-0.007
eact_it1	-0.091	-0.036	-0.027	0.901	-0.073	0.023	0.038	0.019
eact_it3	-0.011	-0.002	-0.006	0.916	-0.043	-0.003	-0.115	0.021
eact_it4	0.021	-0.014	0.025	0.795	0.137	-0.003	0.026	-0.020
eact_it5	0.085	0.055	0.008	0.810	-0.025	-0.017	0.060	-0.021
erel_it1	-0.012	0.034	0.044	0.026	0.917	-0.016	0.063	-0.029
erel_it3	0.012	-0.034	-0.044	-0.026	0.939	0.016	-0.063	0.029
int_it1	-0.011	0.042	-0.111	-0.003	0.060	0.854	-0.092	-0.002
int_it2	-0.053	-0.071	0.129	-0.009	-0.086	0.715	0.035	-0.015
int_it3	0.060	0.025	-0.011	0.011	0.021	0.830	0.058	0.016
wom_it1	0.089	-0.071	-0.041	0.056	-0.135	0.070	0.891	0.000
wom_it2	-0.089	0.071	0.041	-0.056	0.135	-0.070	0.881	-0.000
loi_it1	0.030	-0.023	0.083	0.041	-0.134	-0.079	0.119	0.837
loi_it3	-0.044	-0.003	-0.022	0.006	0.072	-0.069	-0.014	0.783
loi_it4	0.009	0.026	-0.063	-0.046	0.068	0.140	-0.106	0.841

b. The fitness of the model

The goodness of fit of a PLS-based model is measured with three indices: the average path coefficient (APC), the average-R squared (ARS) and the average variance inflation factor (AVIF). For assessing the fitness of a model, the APC and ARS indices should have p values lower than 0.05, and secondly, AVIF should be lower than 5 [**23**] (**Table 6**). 

**Table 6:** The fitness indices of the model

The value of the coefficient	Level of statistical significance	The ideal value for validation
APC=0.13	P<0.01	P<0.05
ARS=0.13	P<0.01	P<0.05
AARS=0.11	P<0.02	P<0.05
AVIF=1.12	-	<=3.3
AFVIF=1.17	-	<=3.3

Since the validity, the reliability and the fitness of the model have proved that it is correctly defined, the hypotheses of the model using structural equation modeling were investigated.

c. Structural Equation Modeling 

**Fig. 2:**
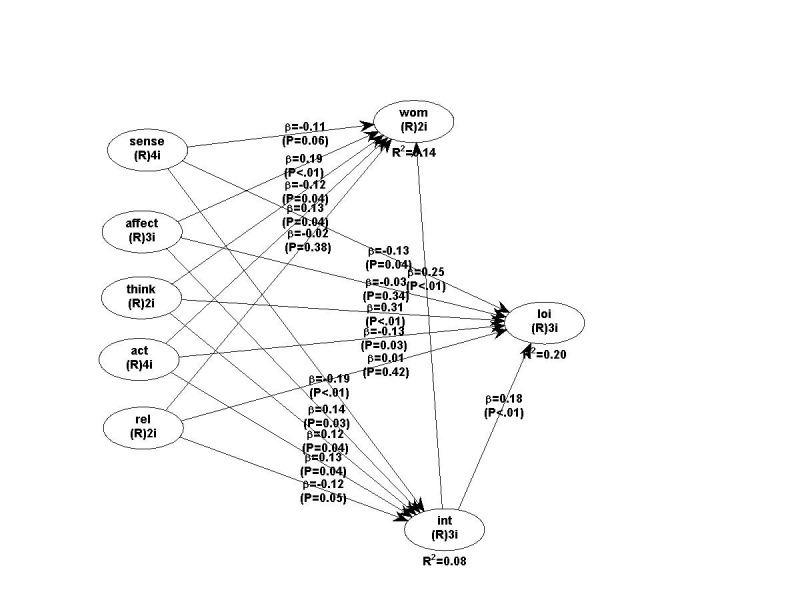
The resulted model in Warp PLS

**Fig. 3:**
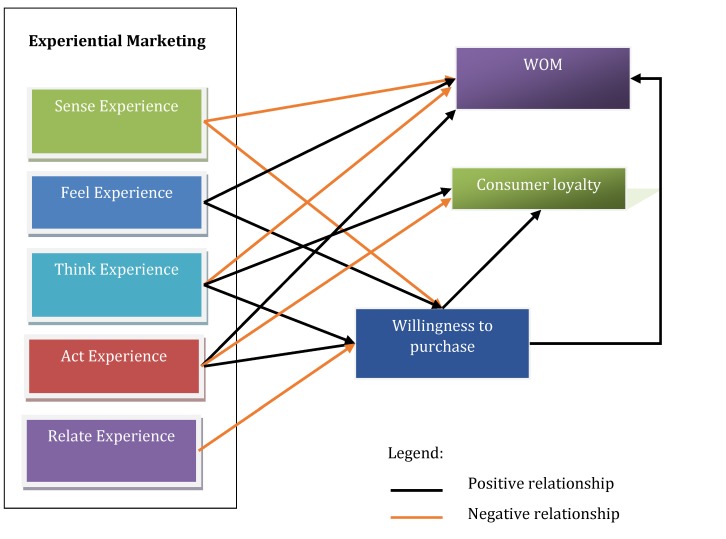
The refined model

The PLS-Structural Equation Modeling is based on the Partial Least Squares method and measures the connection between latent variables using beta standardize d coefficients and R square values. The PLS-SEM model, the path coefficients, their associated p values and the hypotheses’ testing are illustrated in **Table 7** and **Fig. 2**. A refined model is presented in **Fig. 3**. 

**Table 7:** Hypotheses’ statuses

No.	Hypotheses	β	Level of statistical significance	Validation
H1	Sense experience -> WOM	-0.11	0.056	NO
H2	Sense experience -> consumer loyalty	-0.12	0.037	YES
H3	Sense experience -> willingness to purchase	-0.19	0.003	YES
H4	Feel experience -> WOM	0.18	0.004	YES
H5	Feel experience -> consumer loyalty	-0.03	0.336	NO
H6	Feel experience -> willingness to purchase	0.13	0.027	YES
H7	Think experience -> WOM	-0.12	0.045	YES
H8	Think experience -> consumer loyalty	0.31	<0.001	YES
H9	Think experience -> willingness to purchase	0.12	0.041	YES
H10	Act experience -> WOM	0.12	0.036	YES
H11	Act experience -> consumer loyalty	-0.12	0.034	YES
H12	Act experience -> willingness to purchase	0.12	0.039	YES
H13	Relate Experience -> WOM	-0.02	0.383	NO
H14	Relate experience -> consumer loyalty	0.01	0.422	NO
H15	Relate experience-> willingness to purchase	-0.12	0.046	YES
H16	Willingness to purchase -> WOM	0.24	<0.001	YES
H17	Willingness to purchase -> consumer loyalty	0.18	0.005	YES

The R2 coefficient for willingness to purchase construct was of 0.08, meaning that the experiential marketing components explain 8% of its variance, which is classified as acceptable, whereas the R2 coefficient for the WOM construct was of 0.14, meaning that the experiential marketing constructs and the willingness to purchase a private ophthalmologic service explain 14% of its variance, and the consumer loyalty construct is explained in a proportion of 20% by experiential marketing and the willingness to purchase. Overall, the model proved to be satisfactory in the context of private ophthalmologic services. 

## Conclusions

The respondents who appreciated the application of experiential marketing in ophthalmologic services were 71.05%, 18.95% being confused. Moreover, the participants’ demographic profile was the following: age between 36 and 45 years, females with college studies and with a frequency of ophthalmologic consultations at every 3 months. 

Further, there were 89.63% of the respondents who appreciated the application of experiential marketing in ophthalmologic services and also expressed their willingness of buying the service. 

The design of a model containing both the constituent elements of the experimental marketing and its consequences in ophthalmology services was conducted by modeling with structural equations in WarpPLS version 6.0 software. Thus, the validity of the model was assessed with the Cronbach’s alpha coefficients, Composite reliability values, as well as with the Average Variance Extracted coefficients and the fitness of the model was determined by using the ARS, APC, and AVIF values, respectively. 

According to beta coefficients and levels of statistical significance, some hypotheses have been rejected or negative relationships have been established between dependent and independent variables. Therefore, negative relationships have been established between sensory experience and consumer loyalty, as well as sensory experience and willingness to purchase an ophthalmologic service. One possible explanation would be that an ophthalmologic consultation does not imply a high degree of customization, the vast majority of ophthalmic organizations offer specific services under excellent conditions, and consumers no longer value the environmental elements but the perceived value of the service. Further, consumers are no longer attracted by technical and technological equipment but by other elements that give them a sense of respect, integrity, and dignity, as well as by empowering them to take part in the health decision-making process. 

Moreover, affective experience influences the consumer of ophthalmic services at the level of purchasing intention and generating recommendations. The health service remains a typology that requires a lot of patient confidence but also he perceives very high risks, such as psychological risk through fear, social risk through manifestation of social aversion and marginalization of sick people, physical risk, and of course, the functional risk, namely the long waiting for the outcome to be observed. All these risks may be solved if the physician adopts an empathic attitude, as consumers of ophthalmologic services lack the necessary knowledge of understanding the specialized language. An appropriate attitude of the physician will generate a positive intention to purchase the ophthalmologic service and would raise the level of recommending the service to relatives and peers as well as on the Internet in virtual patient communities.

It is difficult to position on the ophthalmology market in Romania, as the vast majority of consumers choose the service provider according to the location. In these circumstances, the ophthalmologist managers can successfully use experimental marketing strategies if they promote a story, meaning a life experience.
